# IGF2BP2 acts as a m^6^A modification regulator in laryngeal squamous cell carcinoma through facilitating CDK6 mRNA stabilization

**DOI:** 10.1038/s41420-023-01669-7

**Published:** 2023-10-10

**Authors:** Xiaojun Tang, Qinglai Tang, Shisheng Li, Mengmeng Li, Tao Yang

**Affiliations:** Department of Otolaryngology Head and Neck Surgery, The Second Xiangya Hospital of Central South University, Changsha, Hunan China

**Keywords:** Head and neck cancer, Tumour biomarkers

## Abstract

Laryngeal squamous cell carcinoma (LSCC) is one of the most commonly seen cancers in the head and neck region with increasing morbidity and mortality globally. N6-methyladenosine (m^6^A) modification plays a critical role in the carcinogenesis of LSCC. In this study, two datasets from online database were analyzed for differentially expressed genes (DEGs) between LSCC and normal samples. Furthermore, we carried out a series of experiments, including hematoxylin & eosin staining, immunohistochemical (IHC) staining, CCK-8, colony formation, transwell, flow cytometry, xenograft tumor model assays, actinomycin D assay, cycloheximide (CHX) assay, methylated m^6^A RNA immunoprecipitation (Me-RIP), RNA immunoprecipitation (RIP) assay, to verify the relevant findings in vivo and in vitro. Insulin like growth factor 2 mRNA binding protein 2 (IGF2BP2) was identified as an up-regulated m^6^A regulator in LSCC samples. Lower IGF2BP2 expression was linked to higher survival probability in LSCC and other head and neck squamous cell carcinoma patients. In LSCC cells, IGF2BP2 knockdown attenuated cancer cell aggressiveness, possibly through modulating cell cycle arrest. In the xenograft tumor model derived from IGF2BP2 knocked-down LSCC cells, IGF2BP2 knockdown inhibited tumor growth. IGF2BP2 up-regulated CDK6 expression through facilitating the stability of CDK6 mRNA and protein. CDK6 knockdown caused no changes in IGF2BP2 expression, but partially eliminated the promotive effects of IGF2BP2 overexpression on LSCC cells’ aggressiveness. Overexpressed IGF2BP2 in LSCC serves as an oncogenic factor, promoting LSCC cell proliferation and invasion in vitro and tumor growth in a xenograft tumor model in vivo through facilitating CDK6 mRNA stabilization.

## Introduction

Laryngeal squamous cell carcinoma (LSCC) is one of the most commonly seen cancers in the head and neck region, which accounts for more than 95% of all laryngeal malignant tumors [1]. The 5-year survival rates for most malignancies have increased as diagnostic and therapeutic techniques have evolved, but the 5-year survival rate for laryngeal cancer has decreased over the last 40 years [2]. According to cancer statistics in China and United States, the morbidity and mortality of laryngeal carcinoma are increasing globally [3, 4].

N6-methyladenosine (m^6^A) is the most common modification in RNAs, first discovered on mRNA and later on non-coding mRNAs such as circRNAs and lncRNAs [5, 6]. m^6^A modification could influence the fate of modified RNAs via influencing posttranscriptional modulation including RNA stability, splicing, and translational efficiency, which affects a variety of biological processes such as cell differentiation, tissue development, spermatogenesis, and RNA-protein crosstalks [7, 8]. Therefore, m^6^A RNA methylation also exerts a crucial effect on numerous human illnesses. For example, Chen et al. [9] discovered that the involvement of WTAP in m^6^A methylation exerts an important effect on hepatocellular carcinoma progression. Moreover, Wang et al. [10] discovered that increased METTL3 expression enhances gastric tumor angiogenesis/glycolysis. Bai et al. [11] discovered that YTHDF1 exerts an important oncogenic effect on colorectal cancer via enhancing Wnt/β-catenin signaling. Nevertheless, the RNA m^6^A expression and patterns and the underlying mechanisms in LSCC have not been fully elucidated yet.

Herein, differentially expressed genes between LSCC and normal samples were analyzed using online datasets and differentially expressed m^6^A regulators were identified. The in vitro and in vivo effects of candidate m^6^A regulator were investigated using LSCC cell lines and xenograft tumor model in nude mice. Regarding molecular mechanism, the downstream factor that might be modulated by the candidate m^6^A regulator was analyzed and validated.

## Results

### Integrative bioinformatics analysis identifying key genes affecting LSCC progression

The GSE59102 microarray dataset included the expression profiles of 12 cases of normal (surgical margins) and 29 cases of LSCC tissues, and the detection platform was GPL6480, which included 50636 probes targeting 23080 genes; the difference matrix was obtained after the limma package homogenization, yielding 3140 down-regulated and 3569 up-regulated genes with a threshold of logfc >0.6 or <−0.6, *p* < 0.05 (Fig. S1A). The GSE143224 microarray dataset included 17 normal (surgical margins) and 17 LSCC tissues, and the detection platform was GPL571, which included 316919 probes targeting 17342 genes; the difference matrix was obtained after the limma package homogenization, yielding 1130 down-regulated and 852 up-regulated genes with a threshold of logfc >0.6 or <−0.6, *p* < 0.05 (Fig. S1B). After comparison, a total of 491 up-regulated and 516 down-regulated genes were overlapped (Fig. S1C, D). Overlapped deregulated genes (up- and down-regulated) were applied for GO functional and Kyoto Encyclopedia of Genes and Genomes (KEGG) signaling pathway enrichment annotation analyses. Based on GO analysis, up-regulated genes were functionally linked to viral infection and response, cell nuclear replication, and outer matrix remodeling, all important processes in LSCC; based on KEGG analysis showed that up-regulated genes were primarily linked to HPV viral infection, cell cycle, and ECM receptor interactions (Fig. S1E). Based on GO analysis, down-regulated genes were linked to lipid metabolism in LSCC; based on KEGG analysis, down-regulated genes were primarily linked to lipid metabolic pathway processes, suggesting that normal digestive tract functions were lost after carcinogenesis (Fig. S1F).

### IGF2BP2 is up-regulated in LSCC

**Fig. 1:**
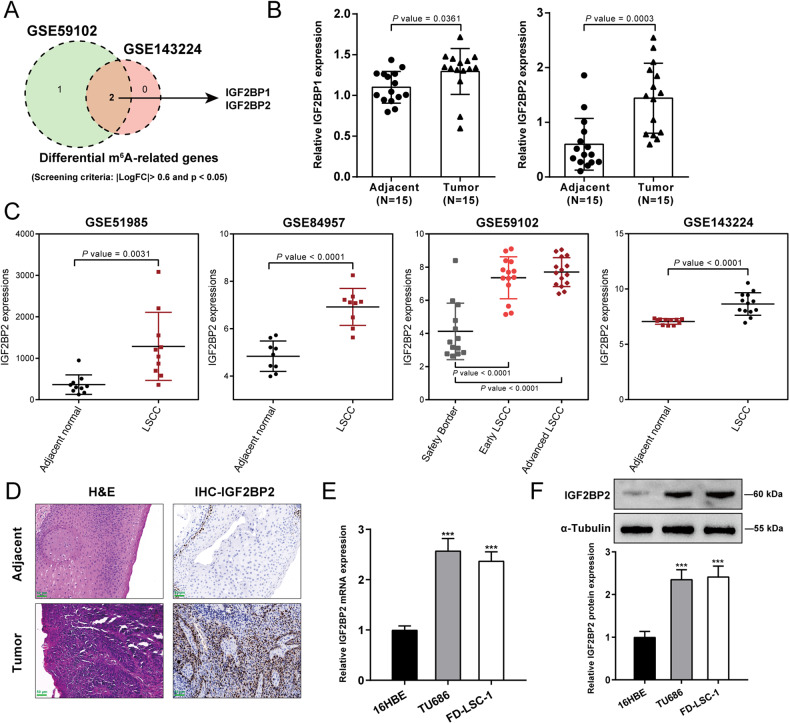
IGF2BP2 is up-regulated in LSCC. **A** Differentially expressed m^6^A-associated genes between LSCC and non-cancerous control samples were analyzed and compared based on GSE59102 and GSE143224. **B** The expression levels of IGF2BP1 and IGF2BP2 were examined in LSCC and non-cancerous control samples using qRT-PCR. **C** The expression levels of IGF2BP2 in LSCC and non-cancerous control samples according to GSE51985, GSE84957, GSE59102, and GSE143224. **D** The histopathological characteristics and IGF2BP2 levels in LSCC and non-cancerous control samples were analyzed using H&E and Immunohistochemical (IHC) staining, respectively. Scale bar = 50 μm. **E**, **F** The mRNA expression and protein levels of IGF2BP2 were examined in normal and LSCC cells using qRT-PCR and Immunoblotting, respectively. ***P < 0.001 compared to 16HBE cells.

As aforementioned, m^6^A RNA modification exerts a critical effect on the carcinogenesis of head and neck squamous cell carcinoma [12]. Therefore, differentially expressed m^6^A-associated genes between LSCC and non-cancerous control samples were analyzed and compared based on GSE59102 and GSE143224; IGF2BP1 and IGF2BP2 were up-regulated in LSCC samples according to both datasets (Fig. 1A). In collected LSCC samples, IGF2BP2 were more up-regulated compared with non-cancerous control samples (Fig. 1B). According to GSE51985, GSE84957, GSE59102, and GSE143224, IGF2BP2 expression showed to be significantly up-regulated in LSCC compared with those in non-cancerous control samples (Fig. 1C). According to GSE31056, GSE20347, and GSE138206, the expression levels of IGF2BP2 were considerably up-regulated in other head and neck squamous carcinomas compared with those in non-cancerous control samples (Fig. S2A). According to TCGA-HNSC, IGF2BP2 was significantly up-regulated in HNSC compared with non-cancerous control samples and increased with the T stages progressing (Fig. S2B). In collected LSCC samples, IHC staining also indicated higher IGF2BP2 levels than normal control (Fig. 1D). Consistently, IGF2BP2 mRNA and protein expression showed to be dramatically up-regulated within TU686 and FD-LSC-1 cell lines as compared to those within a normal cell line 16HBE (Fig. 1E, F). Based on cases with head and neck squamous cell carcinomas from KMPLOTS database, the prognostic value of IGF2BP2 was analyzed and lower IGF2BP2 expression was linked to higher survival probability in head and neck squamous cell carcinoma patients (Fig. S2C). Therefore, the in vitro and in vivo effects of IGF2BP2 upon LSCC were further investigated.

### Specific effects of IGF2BP2 knockdown on cancer cell aggressiveness

**Fig. 2:**
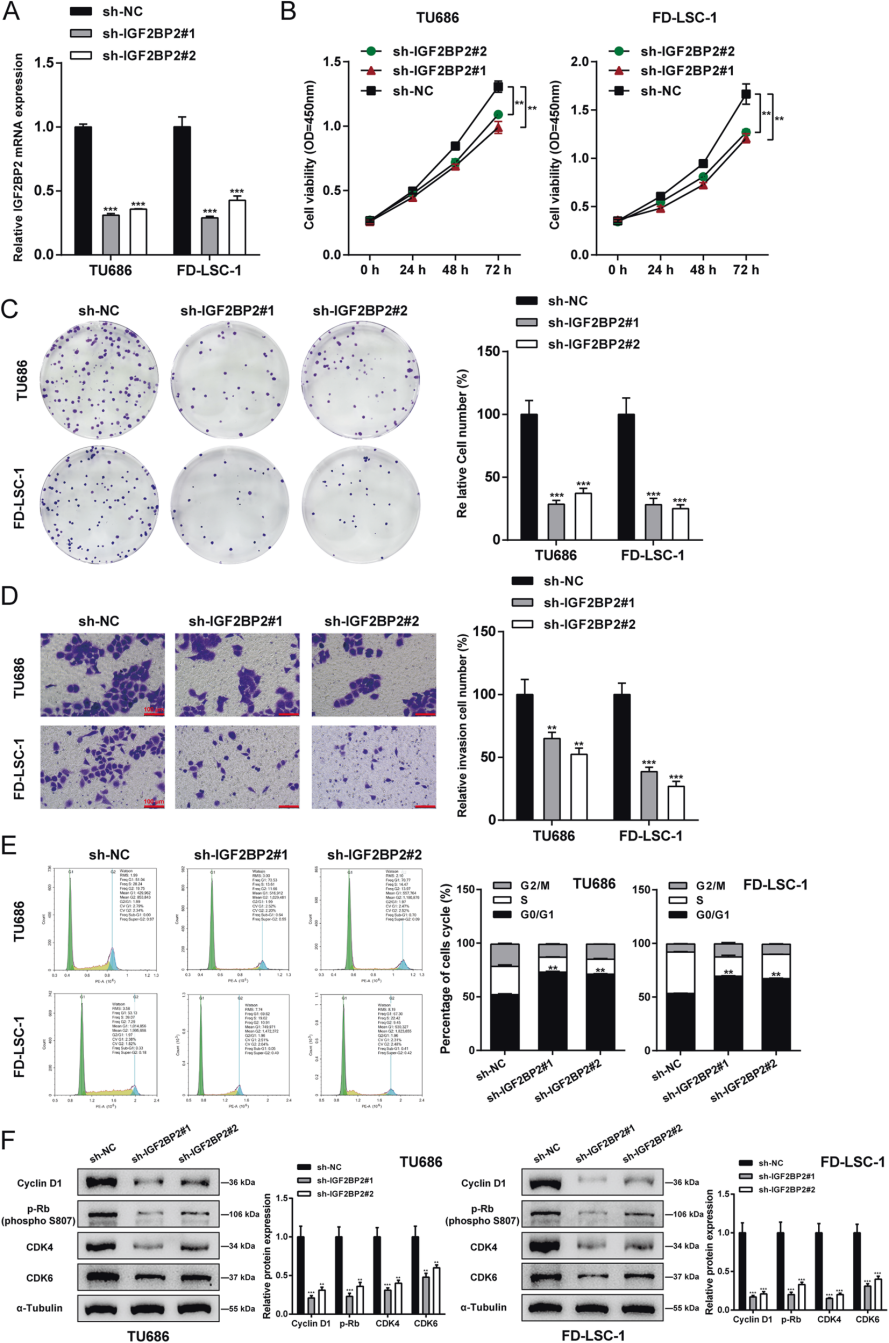
Specifc effects of IGF2BP2 knockdown on cancer cell aggressiveness. **A** IGF2BP2 knockdown was achieved in target cells by transducing short hairpin RNA targeting IGF2BP2 (sh-IGF2BP2#1/2); IGF2BP2 knockdown was verified using qRT-PCR. TU686 and FD-LSC-1 cells were transduced with sh-IGF2BP2#1/2 and examined for cell viability by CCK-8 assay (**B**); colony formation capacity (**C**); cell invasion by Transwell assay (**D**); cell cycle by Flow cytometry assay (**E**); the protein levels of cyclin D1, p-Rb (S807), CDK4, and CDK6 by Immunoblotting (**F**). **P < 0.01, ***P < 0.001 compared to sh-NC group.

Considering higher IGF2BP2 expression in TU686 and FD-LSC-1 cells, IGF2BP2 knockdown was achieved in target cells by transducing sh-IGF2BP2#1/2 and IGF2BP2 knockdown was verified using qRT-PCR (Fig. 2A). IGF2BP2 knockdown in TU686 and FD-LSC-1 cell lines remarkably suppressed cancer cell viability (Fig. 2B), colony formation capacity (Fig. 2C), and cell invasion (Fig. 2D) and elicited G0/1-phase arrest of cell cycle (Fig. 2E). The protein contents of cell cycle checkpoint markers, cyclin D1, p-Rb, CDK4, and CDK6 were examined by Immunoblotting. Figure 2F shows that IGF2BP2 knockdown obviously reduced cyclin D1, p-Rb, CDK4, and CDK6 proteins. These findings suggest that IGF2BP2 knockdown attenuates the aggressiveness of LSCC cells, possibly through modulating cell cycle arrest.

### In vivo effects of IGF2BP2 knockdown on xenograft tumor model in nude mice

**Fig. 3:**
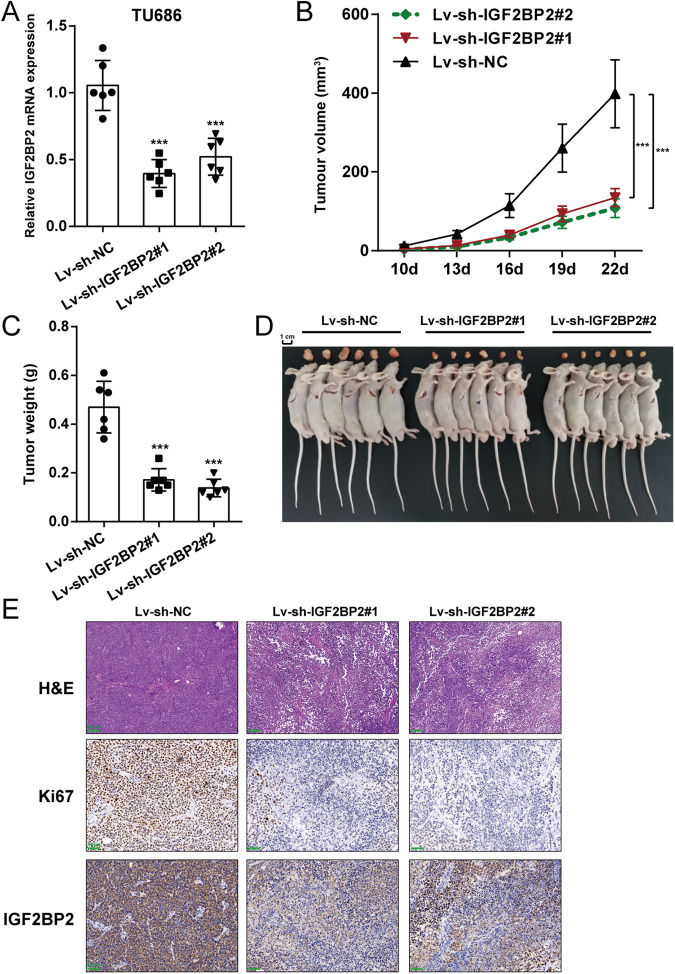
In vivo effects of IGF2BP2 knockdown on xenograft tumor model in nude mice. **A** TU686 cells were transduced with sh-IGF2BP2#1/2 and examined for IGF2BP2 knockdown by qRT-PCR. **B** TU686 cells transduced with sh-IGF2BP2#1/2 were injected into nude mice for establishing a xenograft tumor model; the tumor volumes were evaluated from day 10 of the injection every three days. Mice were anesthetized and euthanized on day 25 of the injection, tumors were removed and collected, tumor weight was determined (**C**, **D**) and histopathological examinations were performed (**E**). Scale bar = 100 or 50 μm. ***P < 0.001 compared to lv-sh-NC group.

Regarding the in vivo effects of IGF2BP2 knockdown, IGF2BP2 knocked-down LSCC cells were used for the establishment of a xenograft tumor model in nude mice. TU686 cells were transduced with sh-IGF2BP2#1/2 and examined for IGF2BP2 knockdown by qRT-PCR (Fig. 3A). Transduced TU686 cells were injected into nude mice for establishing a xenograft tumor model; the tumor volumes were evaluated from day 10 of the injection every 3 days. Figure 3B shows that IGF2BP2 knockdown gradually decreased the tumor volumes compared with tumors derived from Lv-sh-NC-transduced TU686 cells. On day 25 of the injection, tumors were removed and collected. IGF2BP2 knockdown significantly decreased tumor weight compared with tumors derived from Lv-sh-NC-transduced TU686 cells (Fig. 3C, D). Histopathological examinations further indicated that IGF2BP2 knockdown significantly decreased the levels of Ki67 and IGF2BP2 in tumor tissues compared with tumors derived from Lv-sh-NC-transduced TU686 cells (Fig. 3E).

### IGF2BP2 mediates the stability and expression of CDK6

**Fig. 4:**
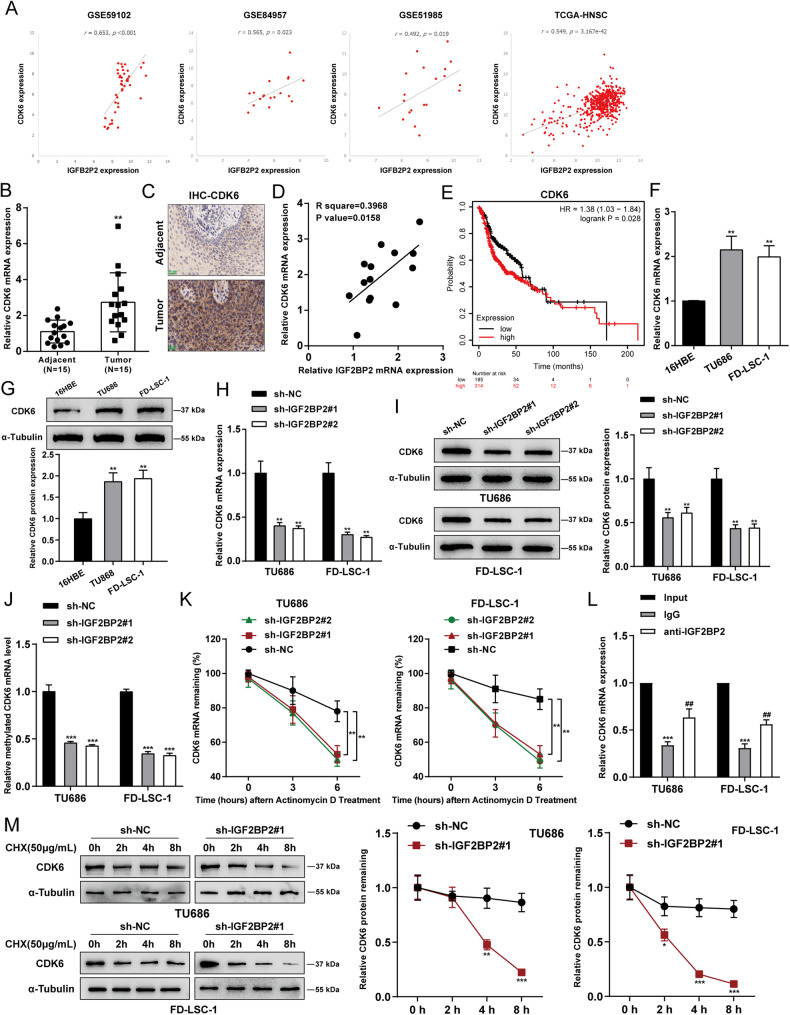
IGF2BP2 mediates the stability and expression of CDK6. **A** The correlation between IGF2BP2 and CDK6 expression in tissue samples according to GSE59102, GSE84957, GSE51985, and TCGA-HNSC. **B** The mRNA expression of CDK6 was examined in collected cancer and normal control samples using qRT-PCR.**P < 0.01 compared to normal control samples. **C** The protein levels of CDK6 were examined in collected cancer and normal control samples using IHC staining. Scale bar = 20 μm. **D** The correlation between IGF2BP2 and CDK6 expression in tissue samples was analyzed using Pearson’s correlation analysis. **E** The association between CDK6 expression and the overall survival of head and neck squamous carcinoma patients was analyzed based on data from KMPLOTS database. **F**, **G** The mRNA expression and protein levels of CDK6 were examined in normal and LSCC cells using qRT-PCR and Immunoblotting, respectively. **P < 0.01 compared to 16HBE cells. **H**, **I** The mRNA and protein levels of CDK6 were detected in IGF2BP2-silenced FD-LSC-1 and TU686 cells by qRT-PCR and Western blotting, respectively. **J** The methylated CDK6 mRNA level in the IGF2BP2-silenced FD-LSC-1 and TU686 cells were analyzed by Me-RIP assay. **K** The levels of CDK6 mRNA were examined by qRT-PCR after IGF2BP2-silenced FD-LSC-1 and TU686 cells were treated with actinomycin D. **P < 0.01 compared to sh-NC group. **L** RIP assay was applied in FD-LSC-1 and TU686 cells to determine the combined relationship between IGF2BP2 and CDK6. ***P < 0.001 compared to Input group; ## *P* < 0.01 compared to IgG group. **M** CDK6 protein stability in IGF2BP2-silenced FD-LSC-1 and TU686 cells were detected using CHX assay. *P < 0.05, **P < 0.01, ***P < 0.001 compared to sh-NC group.

Considering that the cell cycle plays an important role in cancer development [13, 14], and previous experiments found that IGF2BP2 regulated cell cycle checkpoint markers expression in LSCC cells. Gene Expression Profiling Interactive Analysis (GEPIA, http://gepia.cancer-pku.cn/index.html) was applied to analyze the expression correlation between IGF2BP2 and cyclin D1, CDK4, or CDK6 based on TCGA-HNSC dataset (Fig. S3). Among them, the expression of IGF2BP2 was most correlated with CDK6 (*P* = 9.4 × 10^−43^). Therefore, CDK6 was selected as a follow-up research object. Furthermore, according to GSE59102, GSE84957, GSE51985, and TCGA-HNSC, IGF2BP2 and CDK6 expression were significantly positively correlated in tissue samples (Fig. 4A). In collected LSCC samples, CDK6 mRNA and protein expression showed to be dramatically up-regulated than normal control (Fig. 4B, C). In collected tissue samples, IGF2BP2 was also significantly positively correlated with CDK6 expression (Fig. 4D). Based on data from KMPLOTS database, lower expression of CDK6 was linked to higher survival probability in head and neck squamous carcinoma patients (Fig. 4E). Similar to IGF2BP2, CDK6 mRNA expression and protein levels were remarkably up-regulated in TU686 and FD-LSC-1 cell lines than those in normal cell lines (Fig. 4F, G). Furthermore, in TU686 and FD-LSC-1 cell lines transduced with sh-IGF2BP2#1/2, CDK6 mRNA expression and protein levels were also significantly down-regulated (Fig. 4H, I). As revealed by Me-RIP assay, IGF2BP2 knockdown reduced methylated CDK6 mRNA levels (Fig. 4J). Moreover, within FD-LSC-1 and TU686 cell lines, actinomycin D inhibition of RNA synthesis assays showed that CDK6 mRNA levels showed to be down-regulated upon the knockdown of IGF2BP2, indicating that CDK6 mRNA half-life period became shorter upon knockdown of IGF2BP2 (Fig. 4K). The RIP assay was applied to investigate whether IGF2BP2 was physically associated with CDK6 mRNA in FD-LSC-1 and TU686 cells. IGF2BP2 could bind to CDK6 mRNA directly (Fig. 4L). Finally, cells were treated with cycloheximide (CHX) to examine whether IGF2BP2 could affect CDK6 protein stability. The half-life period of CDK6 was significantly reduced by knockdown of IGF2BP2 in FD-LSC-1 and TU686 cells, indicating a change in its protein stability (Fig. 4M). Collectively, IGF2BP2 appears to upregulate the CDK6 stability by increasing CDK6 mRNA expression via promoting its methylation.

### IGF2BP2 promotes LSCC cell aggressiveness through CDK6

**Fig. 5:**
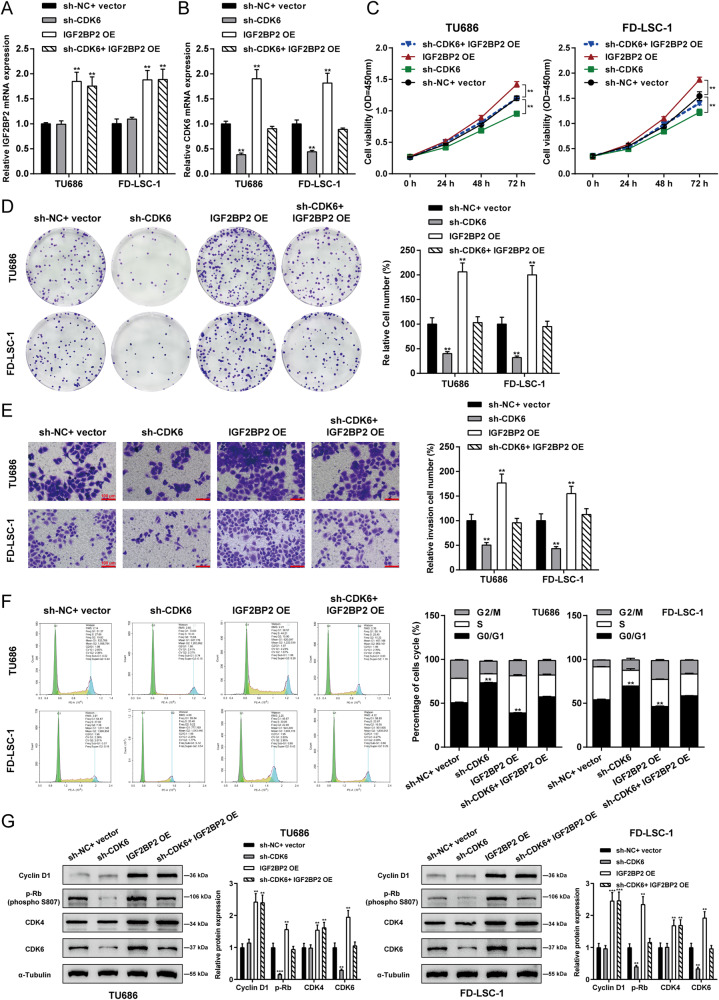
IGF2BP2 promotes LSCC cell aggressiveness through CDK6. Target cells were co-transduced with sh-CDK6 and IGF2BP2 OE and examined for the mRNA expression of CDK6 and IGF2BP2 using qRT-PCR (**A**, **B**); cell viability by CCK-8 assay (**C**); colony formation capacity (**D**); cell invasion by Transwell assay (**E**); cell cycle by Flow cytometry assay (**F**); the protein levels of cyclin D1, p-Rb (S807), CDK4, and CDK6 by Immunoblotting (**G**). **P < 0.01, ***P < 0.001 compared to sh-NC+ vector group.

Last, the dynamic effects of IGF2BP2 and CDK6 on LSCC cell aggressiveness were investigated. Target cells were co-transduced with sh-CDK6 and IGF2BP2 OE and determined for CDK6 and IGF2BP2 mRNA expression levels. As inferred from Fig. 5A, B, sh-CDK6 transduction caused no changes in IGF2BP2 expression but significantly down-regulated CDK6 expression, whereas IGF2BP2 OE transduction dramatically up-regulated IGF2BP2 and CDK6 expression levels; co-transduction of sh-CDK6 and IGF2BP2 OE caused no changes in IGF2BP2 expression but significantly attenuated the promotive effects of IGF2BP2 OE on CDK6 expression. Regarding cellular functions, CDK6 knockdown also significantly inhibited cell viability (Fig. 5C), colony formation ability (Fig. 5D), and cell invasion (Fig. 5E) and elicited G0/1-phase arrest of cell cycle (Fig. 5F), whereas IGF2BP2 overexpression aggravated cancer cell aggressiveness (Fig. 5C–F). Furthermore, the oncogenic effects of IGF2BP2 overexpression were partially eliminated by CDK6 knockdown (Fig. 5C–F). Consistently, IGF2BP2 overexpression increased cyclin D1, p-Rb (S807), CDK4, and CDK6 proteins; whereas CDK6 knockdown decreased p-Rb (S807) and CDK6 proteins; the promotive effects of IGF2BP2 overexpression on these markers were partially abolished by CDK6 knockdown (Fig. 5G).

## Discussion

In this study, two datasets from GEO were analyzed for DEGs between LSCC and normal samples, and IGF2BP2 was identified as an up-regulated m^6^A regulator in LSCC samples. Lower IGF2BP2 expression was linked to higher survival probability in LSCC and other head and neck squamous cell carcinoma patients. In LSCC cells, IGF2BP2 knockdown attenuated cancer cell aggressiveness, possibly through modulating cell cycle arrest. In the xenograft tumor model derived from IGF2BP2 knocked-down LSCC cells, IGF2BP2 knockdown inhibited tumor growth. IGF2BP2 up-regulated CDK6 expression through facilitating the stability of CDK6 mRNA and protein. CDK6 knockdown caused no changes in IGF2BP2 expression, but partially eliminated the promotive effects of IGF2BP2 overexpression on LSCC cells’ aggressiveness.

As previously reported, the m^6^A modification was linked to tumorigenesis, tumor growth and tumor cell chemo-resistance [15–17]. Three types of enzymes are involved in m^6^A RNA modification: methyltransferases (writers), demethylases (erasers), and m^6^A-related binding proteins (readers) [18], which are frequently deregulated in malignancies, such as head and neck squamous cell carcinomas. Therefore, m^6^A regulators serve as promising diagnostic and prognostic markers and therapeutic targets [19]. Regarding LSCC, the presence of m^6^A methyltransferase RBM15 overexpression was confirmed. The level of RBM15 showed to be linked to LSCC development and LSCC patients’ prognosis [20]. ALKBH5 is also overexpressed within LSCC and influences the m^6^A-methylated level of KCNQ1OT1 through modulating specific positions upon KCNQ1OT1, modulating cancer cell proliferation, invasion and metastasis [21]. In this study, two datasets from GEO were analyzed for differentially expressed m^6^A regulators between LSCC and normal samples, and IGF2BP2 was identified as an up-regulated m^6^A regulator in LSCC samples. IGF2BP2 acts as a reader [22] and is also an RNA-binding protein (RBP) exerting a posttranscriptional regulatory effect on mRNA localization, stability, and translation [23]. IGF2BP2 exerts a vital effect on cancer development and might act as a critical biological factor for the prognosis of head and neck squamous cell carcinomas. In this study, lower IGF2BP2 expression was linked to higher survival probability in LSCC and other head and neck squamous cell carcinoma patients, suggesting its potential role in LSCC progression.

In previous studies, the oncogenic roles of IGF2BP2 in head and neck malignancies have been demonstrated. For example, integrative bioinformatics analyses indicated that m^6^A regulatory genes were changed in 41% (205/504) of patients with head and neck squamous carcinomas, of which IGF2BP2 was amplified in 20% (101/504) of patients with head and neck squamous carcinomas and exhibited a positive correlation with its mRNA level. Notably, IGF2BP2 was commonly co-amplified with the most common oncogenes in patients with head and neck squamous carcinomas [24]. Furthermore, IGF2BP2 enhances lymph node metastasis and epithelial-mesenchymal transition of head and neck squamous carcinoma cells through stabilizing slug mRNA m^6^A-dependently [25]. In this study, IGF2BP2 also exerted oncogenic functions in vitro and in vivo. In LSCC cells, IGF2BP2 knockdown attenuated cancer cell aggressiveness by repressing cell proliferation, inhibiting cell invasion, and eliciting G0/1-phase arrest of cell cycle. In xenograft tumor model derived from IGF2BP2 knocked-down LSCC cells, IGF2BP2 knockdown inhibited tumor growth. Consistent with the cell cycle arrest, IGF2BP2 knockdown reduced cyclin D1, p-Rb (S807), CDK4, and CDK6 proteins. The occurrence and progression of cancers are tightly associated with cell cycle diseases [26, 27]. Considering that IGF2BP2 affects the mRNA stabilization of multiple factors, IGF2BP2 might exert its functions on LSCC through cell cycle-associated factors.

As expected, online data and experimental analyses inferred that IGF2BP2 up-regulated CDK6 expression through facilitating the stability of CDK6 mRNA. IGF2BP2 knockdown down-regulated CDK6 mRNA expression and decreased CDK6 protein levels, whereas CDK6 knockdown caused no changes in IGF2BP2 expression, suggesting that CDK6 was downstream of IGF2BP2. Functionally, CDK6 silencing significantly attenuated the promotive effects of IGF2BP2 overexpression upon LSCC cells’ aggressiveness. CDK4/6 phosphorylates retinoblastoma tumor suppressor protein (RB) to regulate cell cycle initiation, thereby inactivating its transcriptional repression function [28, 29]. RB is further hyper-phosphorylated and inactivated by CDK2, which enhances the transition from G1 to S-phase of cell-cycle [30]. Owing to their role in tumor biology, CDK4/6 has been considered as a promising molecular target to pharmacologically activate the RB pathway in different cancer types [31]. In this study, through promoting CDK6 mRNA stability, IGF2BP2 increased CDK6 and p-Rb levels, therefore relieving the cell cycle arrest in G0/1 phase and promoting cancer cell proliferation. After knocking down IGF2BP2, CDK6 mRNA stabilization was hindered and cell cycle arrest was detected.

In conclusion, overexpressed IGF2BP2 in LSCC serves as an oncogenic factor, promoting LSCC cell proliferation and invasion in vitro and tumor growth in a xenograft tumor model in vivo through facilitating CDK6 mRNA stabilization.

## Materials and methods

### Bioinformatics analysis

The microarray expression data have been deposited in the Gene Expression Omnibus (GEO, https://www.ncbi.nlm.nih.gov/geo/) of the National Center of Biotechnology Information with accession numbers GSE59102, GSE143224 [32], GSE51985 [33], GSE84957 [34], GSE31056 [35], GSE20347 [36], and GSE138206. The GSE59102 dataset includes the gene expression profiling of 29 LSCC cancer samples and 13 margin samples were collected from patients undergoing surgical ablation of LSCC. The GSE143224 dataset includes the gene expression profiling of 14 paired LSCC samples and matched nonmalignant mucosa. The GSE51985 dataset includes the gene expression profiling of 10 paired LSCC samples and corresponding adjacent non-neoplastic tissues. The GSE84957 dataset includes the gene expression profiling of 9 pairs of primary Stage IV LSCC tissues and adjacent non-neoplastic tissues. The GSE31056 dataset includes the gene expression profiling of 23 tumor tissues and 73 margins from 24 patients with squamous cell carcinoma of the tongue. The GSE20347 dataset includes the gene expression profiling of 17 paired tumor and matched normal adjacent tissue from 17 esophageal squamous cell carcinoma patients from a high-risk region of China. The GSE138206 dataset includes the gene expression profiling of 6 oral squamous cell carcinoma tissues, six adjacent normal tissues, and six contralateral normal tissues. Differentially expressed genes were screened using Student’s *t* test (*P* value < 0.05) accompanied by |log2 (fold change)| > 0.6 based on GSE59102 and GSE143224 datasets. KEGG signaling and pathway enrichment annotation was performed, and Gene Ontology (Biological Process) functional enrichment annotation was performed using the functional annotation tool of Metascape (https://metascape.org/gp/).

### Clinical tissue sampling

Twenty LSCC tissue samples and twenty adjacent non-cancerous tissue samples were collected from patients that underwent surgical resection at Second Xiangya Hospital with the approval of the Ethic Committee of Second Xiangya Hospital. Written informed consent was obtained from all patients enrolled. All the tissue samples were preserved by snap freezing and kept at −80 °C.

### qRT-PCR

Total RNA was isolated from target tissues or cells using Trizol reagent (Invitrogen, Waltham, MA, USA). The expression levels of target factors were evaluated with a SYBR Green qPCR assay (Takara, Dalian, China) based on the previously described methods [37, 38]. For data processing, the 2^−ΔΔCT^ method was used taking α-tubulin expression as an endogenous control. The primer sequences used in qRT-PCR are listed in Table 1.

**Table 1:** The primer sequence for the study.

	Gene	Primer	Sequence (5'-3')
PCR primer	IGF2BP2	Forward	AGCCTGTCACCATCCATGC
		Reverse	CTTCGGCTAGTTTGGTCTCATC
	IGF2BP1	Forward	CAAAGGAGCCGGAAAATTCAAAT
		Reverse	CGTCTCACTCTCGGTGTTCA
	CDK6	Forward	TCTTCATTCACACCGAGTAGTGC
		Reverse	TGAGGTTAGAGCCATCTGGAAA
	GAPDH	Forward	ACAGCCTCAAGATCATCAGC
		Reverse	GGTCATGAGTCCTTCCACGAT
Vector primers	sh-NC	Forward	/
		Reverse	/
	sh-IGF2BP2#1	Forward	GATCCGGAAGTGATCGTCAGAATTATCTCGAGATAATTCTGACGATCACTTCCTTTTTG
		Reverse	AATTCAAAAAGGAAGTGATCGTCAGAATTATCTCGAGATAATTCTGACGATCACTTCCG
	sh-IGF2BP2#2	Forward	GATCCGCATGATTCTTGAAATCATGCCTCGAGGCATGATTTCAAGAATCATGCTTTTTG
		Reverse	AATTCAAAAAGCATGATTCTTGAAATCATGCCTCGAGGCATGATTTCAAGAATCATGCG
	sh-CDK6	Forward	GATCCGAAACCATAAAGGATATGATGCTCGAGCATCATATCCTTTATGGTTTCTTTTTG
		Reverse	AATTCAAAAAGAAACCATAAAGGATATGATGCTCGAGCATCATATCCTTTATGGTTTCG
	Vector NC	Forward	/
		Reverse	/
	IGF2BP2 (plvx-puro)	Forward	CTACCGGACTCAGATCTCGAGATGATGAACAAGCTTTACATCGGG
		Reverse	GTACCGTCGACTGCAGAATTCTCACTTGCTGCGCTGTGAGG

### Histopathological investigations

Each tissue sample was fixed in 10% buffered formalin and dried in graded ethanol. Paraffin-embedded specimens were sliced into cross-sections at a thickness of 4 μm and stained with hematoxylin-eosin. Pathological images were photographed using an optical microscope (Olympus, Kyoto, Japan).

For Immunohistochemical (IHC) staining, the slices were incubated overnight with anti-IGF2BP2 (CSB-PA908724, Cusabio, Wuhan, China), anti-Ki67 (27309-1-AP, Proteintech, Wuhan, China), or anti-CDK6 (14052-1-AP, Proteintech) at 4 °C overnight. The Vectastain Universal Elite ABC HRP Kit (Vector Laboratories, Burlingame, CA) was used to perform IHC staining. The sections were examined by pathologists in a blinded manner.

### Immunoblotting

After extracting the total protein from target cells, the BCA protein assay kit (Beyotime, Shanghai, China) was employed to determine the protein level. Protein specimens that have been separated by gel electrophoresis were transferred onto PVDF membranes, followed by blocking with 5% nonfat milk for 1 h at ambient temperature. Next, the PVDF membrane was incubated with the following primary antibodies, respectively: anti-IGF2BP2 (CSB-PA908724, Cusabio), anti-cyclin D1 (60186-1-Ig, Proteintech), anti-p-Rb (S807; #9308, Cell Signaling, Danvers, MA, USA), anti-CDK4 (11026-1-AP, Proteintech), anti-CDK6 (14052-1-AP, Proteintech), or anti-α-Tublin (11224-1-AP, Proteintech; endogenous reference).

### Cell lines

Human bronchial epithelial cell line 16HBE (SCC150) was procured from Sigma-Aldrich (St. Louis, MO, USA) and cultivated in Dulbecco’s Modified Minimum Essential Medium (Corning, Manassas, VA, USA) added with 10% FBS (Corning). Human laryngeal carcinoma cell lines FD-LSC-1 were purchased from BeNa culture collection company (China). TU686 cell line was procured from Xiangya Cell Bank (Central South University, Changsha). Cells were cultivated in RPMI1640 media containing 10% heat-inactivated FBS with penicillin/streptomycin at 37 °C in 5% CO_2_.

### Cell transduction

IGF2BP2 knockdown was achieved in target cells by transducing short hairpin RNA targeting IGF2BP2 (sh-IGF2BP2#1/2; GenePharma, Shanghai, China). IGF2BP2 overexpression was achieved in cells by transducing IGF2BP2-overexpressing plasmid (IGF2BP2 OE, GenePharma). CDK6 knockdown was achieved in cells by transducing short hairpin RNA targeting CDK6 (sh-CDK6, GenePharma). Lipofectamine 3000 Reagent (Thermo Fisher Scientific, Waltham, MA, USA) was employed to perform all cell transfection. The sequences of overexpressing vector and shRNA vector are listed in Table 1.

### CCK-8

Cell viability was determined using a CCK-8 kit (Beyotime, Shanghai, China). Cells were planted into 96-well plates at a density of 5 × 10^3^ cells per well following transfection or treatment. Two hours before the evaluation, 20 μl CCK-8 solution was supplemented to each well, followed by incubation with the solution at 37 °C for 2 h. Using a microplate reader, the optical density value was obtained at 450 nm.

### Colony formation

A total of 1 × 10^5^ transduced cells were seeded on 0.6% agarose into 6-well plates. After two weeks of growth in agarose at 37 °C in 5% CO_2_, the colonies were stained with 0.1% crystal violet and then counted. Colonies consisting of more than 50 cells were counted manually.

### Transwell for invasion

For cell invasion investigation, cells were pretreated with mytomycin for 1 h and suspended with serum-free culture medium at a density of 5 × 10^5^ cells/well in an upper chamber. The chamber was pre-covered with Matrigel. The medium containing serum was supplemented into the bottom chambers. Cells were cultured for 48 h at 37 °C. The non-invasive cells in the upper chambers were removed using cotton swabs. Cells invading onto the bottom of the membrane were preserved for 10 min in 100% methanol before being air-dried, stained with crystal violet, and counted using a microscope.

### Flow cytometry for cell cycle

After serum starvation for 24 h, cells were cultured for another 24 h in FBS. Cells were washed twice in precooled PBS before being resuspended in 300 μL of precooled PBS, then fixed overnight in 75% ethanol (chilled). Cells were centrifugated and washed in precooled PBS, and then resuspended in 0.5 ml PI/RNase staining solution (BD PharMingen, San Diego, CA, USA), followed by incubation in the dark at room temperature for 15 min. Within 1 h, flow cytometry was performed to examine the cells (Novocyte, Agilent, USA).

### Methylated m^6^A RNA immunoprecipitation (Me-RIP)

Anti-m^6^A antibody (ab151230, Abcam, Cambridge, MA, USA) was employed to perform the Me-RIP as previously described [39]. qRT-PCR analysis was carried out on methylated RNA to reveal the mRNA expression of methylated COPS5.

### Actinomycin D inhibition of RNA synthesis

TU686 and FD-LSC-1 cells were seeded onto 6-well plates. Up to 60% confluency after 24 h, cells were incubated with Actinomycin D (5 μg/ml) for 0 h, 3 h, and 6 h. Then, cells were harvested for RNA isolation. The mRNA levels of CDK6 were analyzed by qRT-PCR.

### RNA immunoprecipitation (RIP) assay

The relationship between IGF2BP2 protein and CDK6 mRNA was determined using a Magna RIP RNA-Binding Protein Immunoprecipitation Kit (Merck Millipore, Billerica, USA). The antibodies used for the RIP assay included anti-IGF2BP2 and control IgG (Merck Millipore). The coprecipitated RNAs were used for cDNA synthesis and evaluated by qRT-PCR.

### Protein stability analysis

Protein synthesis was inhibited by treatment with CHX at a concentration (25 μg/mL) for 0, 2, 4, and 8 h. Following incubation, the change in CDK6 protein half-life was detected by performing Immunoblotting analysis.

### Xenograft tumor model

Six-week-old male BALB/c nude mice were randomly assigned to three groups (*n* = 6 in each group): Lv-sh-NC, Lv-sh-IGF2BP2#1, and Lv-sh-IGF2BP2#2. Mice in each group received the subcutaneous injection of 1 × 10^6^ cells (0.1 mL) into the upper right flanks of mice. Cells were pre-transduced with sh-NC, sh-IGF2BP2#1, or sh-IGF2BP2#2 for 48 h before the injection. Starting from day 10 of the injection, the tumor volume was measured every 3 days. Mice were anesthetized and euthanized at day 25 of the injection, tumors were removed and collected, tumor weight was determined in a blind manner. The histopathological examinations were performed. All the procedures were approved by the Animal Welfare Ethic Committee of The Second Xiangya Hospital of Central South University.

### Data processing and statistical analysis

Data were analyzed using GraphPad software. The results were expressed as means ± standard deviation (SD). Shapiro–Wilks test was used to explore whether data are normally distributed. Brown-Forsythe test was used for group variances analysis. Comparisons among groups were assessed using ANOVA. Comparisons between groups were assessed using a Student’s *t* test. The significance level was set at *P* < 0.05.

### Supplementary information

Wb original images for checking

Figure S1 legend

Figure S2 legend

Figure S3 legend

Figure S1

Figure S2

Figure S3

## Data Availability

The datasets analyzed and other data during the current study are available from the corresponding author on reasonable request.
