# Supplementary material for: IGF2BP2 acts as a m6A modification regulator in laryngeal squamous cell carcinoma through facilitating CDK6 mRNA stabilization
Source: Cell Death Discov. 2023 Oct 10;9:371. doi: 10.1038/s41420-023-01669-7 (PMC10564923; doi:10.1038/s41420-023-01669-7)
Supplement: Supplementary file 1 — Wb original images for checking [file 41420_2023_1669_MOESM1_ESM.docx]

***Supplementary Figure for Western Blot***

Figure 2H-IGF2BP2


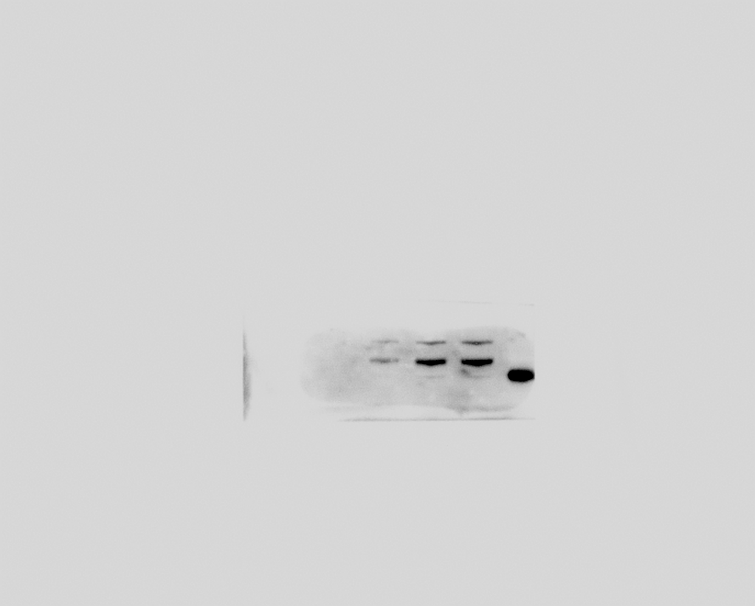


Figure 2H-α-Tubulin





Figure 3F-Cyclin D1 (TU686)





Figure 3F-p-Rb (TU686)





Figure 3F-CDK4 (TU686)





Figure 3F-CDK6 (TU686)





Figure 3F-α-Tubulin (TU686)





Figure 3F-Cyclin D1 (FD-LSC-1)





Figure 3F-p-Rb (FD-LSC-1)





Figure 3F-CDK4 (FD-LSC-1)





Figure 3F-CDK6 (FD-LSC-1)





Figure 3F-α-Tubulin (FD-LSC-1)





Figure 5G-CDK6





Figure 5G-α-Tubulin





Figure 5I-CDK6 (TU686)





Figure 5I-α-Tubulin (TU686)





Figure 5I-CDK6 (FD-LSC-1)





Figure 5I-α-Tubulin (FD-LSC-1)





Figure 6G-Cyclin D1 (TU686)





Figure 6G-p-Rb (TU686)





Figure 6G-CDK4 (TU686)





Figure 6G-CDK6 (TU686)





Figure 6G-α-Tubulin (TU686)





Figure 6G-Cyclin D1 (FD-LSC-1)





Figure 6G-p-Rb (FD-LSC-1)





Figure 6G-CDK4 (FD-LSC-1)





Figure 6G-CDK6 (FD-LSC-1)





Figure 6G-α-Tubulin (FD-LSC-1)
