# Supplementary material for: IGF2BP2 acts as a m6A modification regulator in laryngeal squamous cell carcinoma through facilitating CDK6 mRNA stabilization
Source: Cell Death Discov. 2023 Oct 10;9:371. doi: 10.1038/s41420-023-01669-7 (PMC10564923; doi:10.1038/s41420-023-01669-7)
Supplement: Supplementary file 2 — Figure S1 legend [file 41420_2023_1669_MOESM2_ESM.docx]

**Fig.S1 Integrative bioinformatics analysis identifying key gene affecting laryngeal squamous cell carcinoma (LSCC) progression** (A-B) Differentially expressed genes between LSCC and non-cancerous control samples according to GSE59102 and GSE143224, respectively. (C-D) Overlapped deregulated genes (up- and down-regulated) according to GSE59102 and GSE143224. (E-F) Overlapped deregulated genes (up- and down-regulated) were applied for Gene Ontology (GO) functional and Kyoto Encyclopedia of Genes and Genomes (KEGG) signaling pathway enrichment annotation analyses.
