# Supplementary material for: IGF2BP2 acts as a m6A modification regulator in laryngeal squamous cell carcinoma through facilitating CDK6 mRNA stabilization
Source: Cell Death Discov. 2023 Oct 10;9:371. doi: 10.1038/s41420-023-01669-7 (PMC10564923; doi:10.1038/s41420-023-01669-7)
Supplement: Supplementary file 3 — Figure S2 legend [file 41420_2023_1669_MOESM3_ESM.docx]

**Fig.S2 IGF2BP2 is up-regulated in HNSC** (A) The expression levels of IGF2BP2 in other head and neck squamous carcinomas (HNSC) and non-cancerous control samples according to GSE31056, GSE20347, and GSE138206. (B) The expression levels of IGF2BP2 in HNSC and non-cancerous control samples or HNSC samples in different T stages according to TCGA-HNSC. (C) Cases with head and neck squamous cell carcinomas from the KMPLOTS database (https://kmplot.com/analysis/index.php?p=service&cancer=pancancer_rnaseq) were divided into high- and low-IGF2BP2 groups using the median value as the cutoff and the correlation between IGF2BP2 expression and cancer patients' overall survival was analyzed.
