# Supplementary material for: IGF2BP2 acts as a m6A modification regulator in laryngeal squamous cell carcinoma through facilitating CDK6 mRNA stabilization
Source: Cell Death Discov. 2023 Oct 10;9:371. doi: 10.1038/s41420-023-01669-7 (PMC10564923; doi:10.1038/s41420-023-01669-7)
Supplement: Supplementary file 4 — Figure S3 legend [file 41420_2023_1669_MOESM4_ESM.docx]

**Fig.S3 The expression correlation between IGF2BP2 and CDK6, CDK4 or cyclin D1** Gene Expression Profiling Interactive Analysis (GEPIA, http://gepia.cancer-pku.cn/index.html) was applied to analyse the expression correlation between IGF2BP2 and CDK6 (A), CDK4 (B) or cyclin D1 (C) based on TCGA-HNSC dataset.
