# Supplementary figures and images for: IGF2BP2 acts as a m6A modification regulator in laryngeal squamous cell carcinoma through facilitating CDK6 mRNA stabilization
Source: Cell Death Discov. 2023 Oct 10;9:371. doi: 10.1038/s41420-023-01669-7 (PMC10564923; doi:10.1038/s41420-023-01669-7)

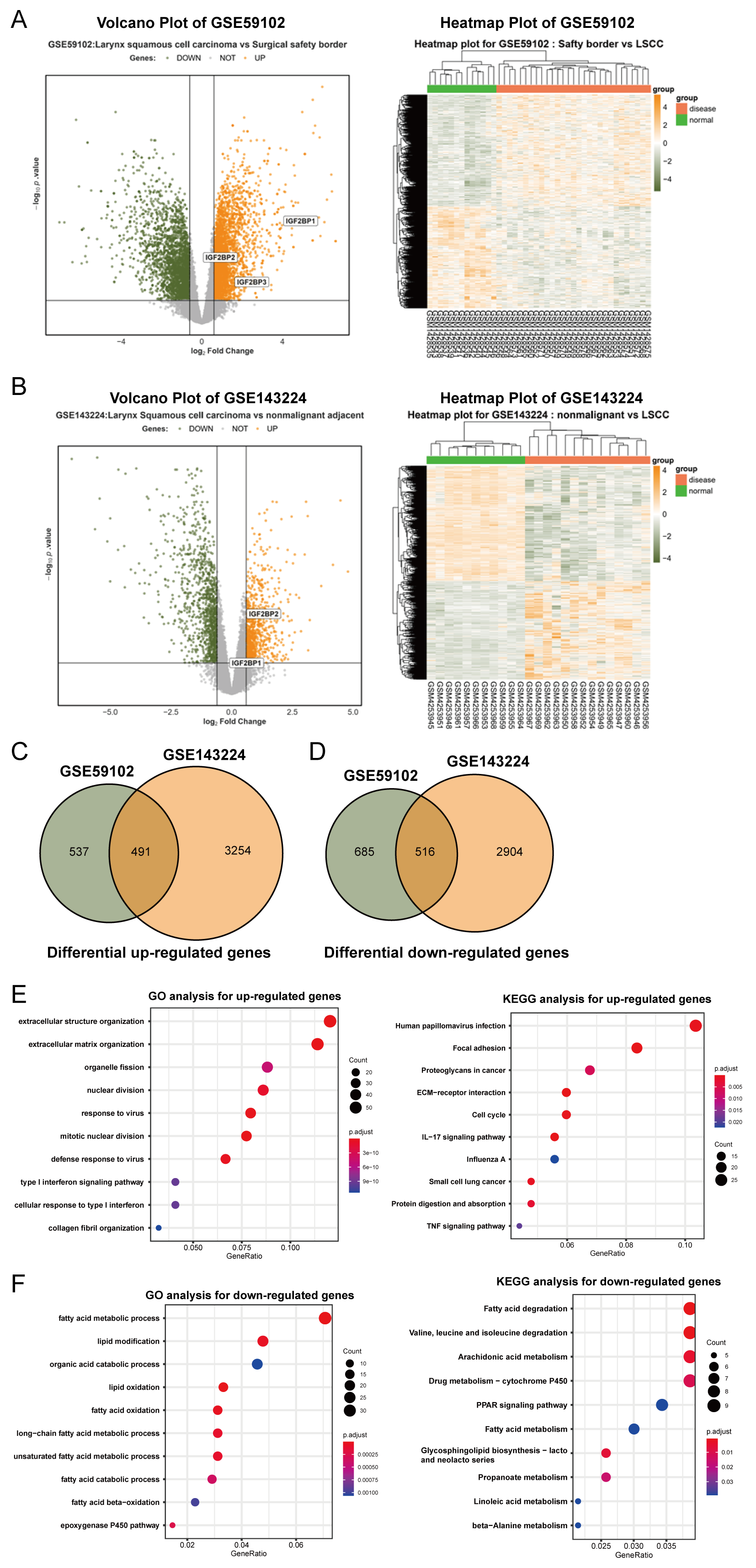

Supplement: Supplementary file 5 — Figure S1 [file 41420_2023_1669_MOESM5_ESM.tif]

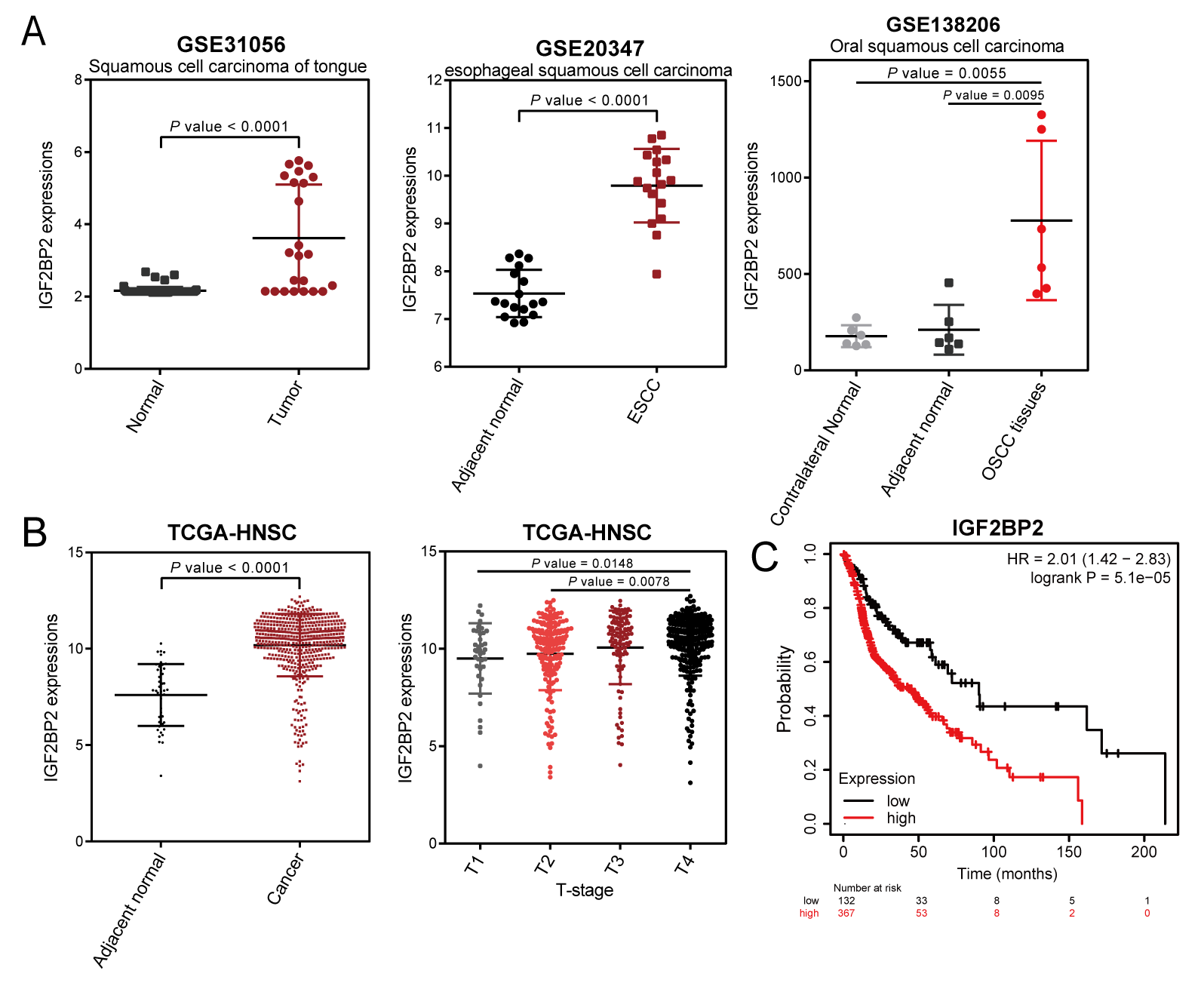

Supplement: Supplementary file 6 — Figure S2 [file 41420_2023_1669_MOESM6_ESM.tif]

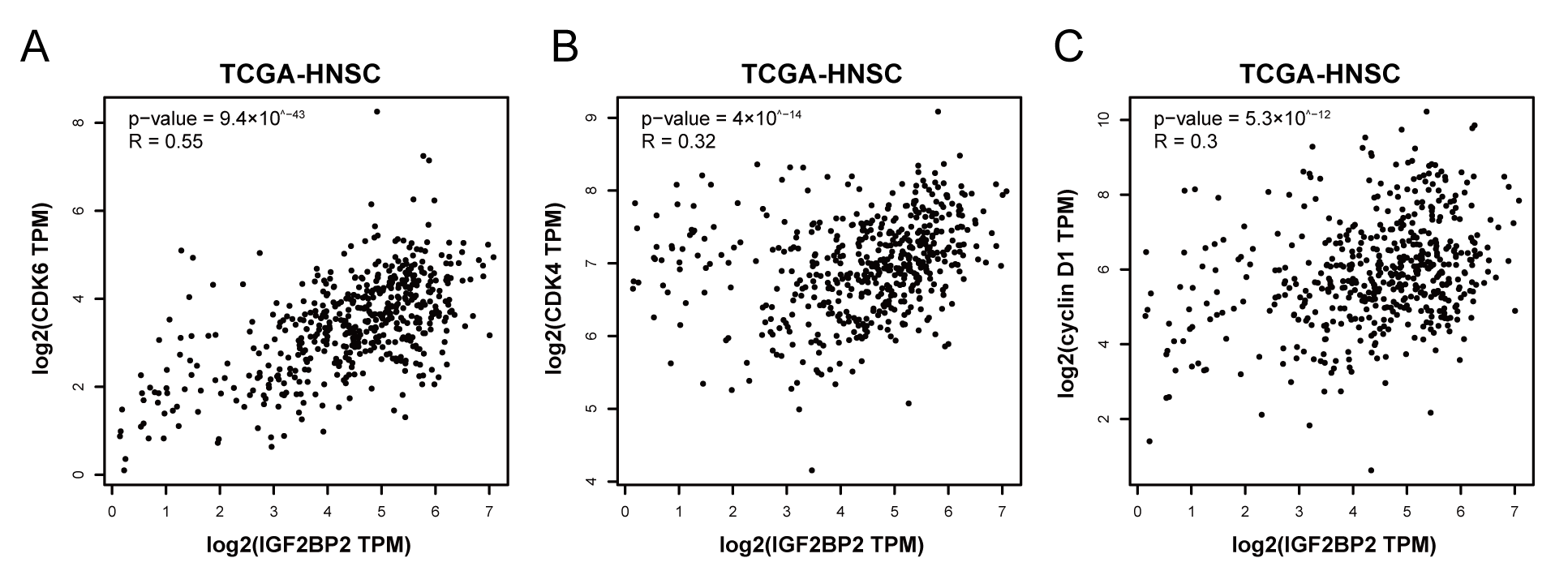

Supplement: Supplementary file 7 — Figure S3 [file 41420_2023_1669_MOESM7_ESM.tif]
